# Neuropeptide Bursicon Influences Reproductive Physiology in *Tribolium Castaneum*

**DOI:** 10.3389/fphys.2021.717437

**Published:** 2021-10-21

**Authors:** Jingjing Li, Zidan Zhu, Jingxiu Bi, Qili Feng, Brenda T. Beerntsen, Qisheng Song

**Affiliations:** ^1^Division of Plant Science and Technology, University of Missouri, Columbia, MO, United States; ^2^Guangdong Key Laboratory of Insect Developmental Biology and Applied Technology, Guangzhou Key Laboratory of Insect Development Regulation and Application Research, Institute of Insect Science and Technology and School of Life Sciences, South China Normal University, Guangzhou, China; ^3^Institution of Quality Standard and Testing Technology for Agro-Product, Shandong Academy of Agricultural Science, Jinan, China; ^4^Department of Veterinary Pathobiology, University of Missouri, Columbia, MO, United States

**Keywords:** *Tribolium castaneum*, bursicon, RNAi, reproduction, vitellogenin, IIS/TOR signaling

## Abstract

Bursicon is a neuropeptide belonging to the cystine knot family and is composed of burs and partner of burs (pburs) subunits. It can form heterodimers or homodimers to execute different biological functions. Bursicon heterodimers regulate cuticle sclerotization and wing maturation, whereas bursicon homodimers mediate innate immunity and midgut stem cell proliferation. A recent study has shown that bursicon potentially induces the expression of vitellogenin (Vg) in the black tiger shrimp *Penaeus monodon*; however, the underlying mechanism remains unknown. In this study, we investigated the role of bursicon in the reproductive physiology of the red flour beetle, *Tribolium castaneum*. The knockdown of burs, pburs, or its receptor *T. castaneum rickets (Tcrk)* in 2-day pupae significantly downregulated the expression levels of Vg1, Vg2, and Vg receptor (VgR) genes in females 3- and 5-day post-adult emergence, leading to abnormal oocytes with limited Vg content. The silencing of burs repressed the number of eggs laid and completely inhibited egg hatch, whereas the silencing of pburs dramatically decreased the number of eggs laid, hatch rate, and offspring larval size, and this RNA interference (RNAi) effects persisted to the next generation. Furthermore, the knockdown of burs or pburs downregulated the expression of the insulin/insulin-like signaling/target of rapamycin (TOR) signaling genes encoding insulin receptor (InR), protein kinase B (Akt), TOR, and ribosomal protein S6 kinase (S6K). Most importantly, the injection of recombinant pburs (r-pburs) protein was able to upregulate the expression of *Vg, VgR, InR, Akt, TOR, S6K, JH synthesis* (*JHAMT*), *Methoprene-tolerant* (*Met*), and *Taiman* (*Tai*) in normal females and rescue the expression of *Vg* and *VgR* in pburs RNAi females but failed to rescue *Vg* and *VgR* in Tcrk knockdown females. We infer that bursicon homodimers influence *Vg* expression *via* the receptor Tcrk, possibly by mediating the expression of the juvenile hormone (JH) and IIS/TOR pathway genes, thereby regulating reproduction in *T*. *castaneum*.

## Introduction

Bursicon is a neuropeptide hormone, which consists of two cystine knot subunits, burs and partner of burs (pburs). It was first discovered in blow flies (Cottrell, 1962) and is mainly responsible for cuticle sclerotization and wing maturation as heterodimers *via* a *Drosophila* leucine-rich repeat G protein-coupled receptor 2 (DLGR2), which is encoded by *rickets* in the fruit fly, *Drosophila melanogaster* (Baker and Truman, 2002; Luo et al., 2005; Mendive et al., 2005). It elicits the cAMP/protein kinase A (PKA) signaling pathway (Luo et al., 2005; Mendive et al., 2005) and further induces the activation of tyrosine hydroxylase, a key enzyme responsible for the synthesis of tanning agents (Davis et al., 2007). In the red flour beetle, *Tribolium castaneum*, bursicon also work through DLGR2 orthologous bursicon receptor encoded by *T. castaneum rickets (Tcrk)* to mediate the postecdysis activity, including elytra, hindwing development, and cuticle sclerotization (Arakane et al., 2008; Hauser et al., 2008; Bai and Palli, 2010). Bursicon can also form homodimers (Honegger et al., 2002). Burs homodimers have been shown to regulate intestinal stem cell proliferation and gut/fat body energy homeostasis in *Drosophila* through DLGR2 (Scopelliti et al., 2014, 2016, 2019). Burs and pburs homodimers have recently been demonstrated to mediate the immune response in *D. melanogaster* and the yellow fever mosquito, *Aedes aegypti*, by upregulating the expression of antimicrobial peptide (AMP) and stress-response genes in the immune deficiency (IMD) pathway in the newly emerged adults when insects are vulnerable to injury and pathogen infection due to unsclerotized cuticle, thereby protecting the body from microbial infection (An et al., 2012; Zhang et al., 2017). A recent report indicates that recombinant bursicon can also regulate vitellogenin (Vg) expression in the black tiger shrimp (Sathapondecha et al., 2015); however, its involvement in *Vg* expression in insects remains unknown.

In insects, Vg is mostly synthesized in the fat body, released into the hemolymph, and taken up by developing oocytes through receptor-mediated endocytosis. After absorption, Vg is stored in the form of vitellin, which is a major part of the nutritional reserve of the developing embryo (Tufail et al., 2014). Therefore, Vg is critical for egg maturation during adulthood and embryonic growth after oviposition. Typically, there are multiple copies of Vg genes encoding multiple Vg proteins in insects to ensure sufficient nutrition for oocyte maturation. The receptor responsible for Vg intake is a membrane-bound protein Vg receptor (VgR) which is another crucial reproduction-related protein in insects in addition to *Vg* (Tufail and Takeda, 2009). Most studies have demonstrated that insect Vg production is generally related to two classic hormones, juvenile hormone (JH) and ecdysteroid hormone, primarily 20-hydroxyecdysone (20E). JH and 20E mediate various aspects of vitellogenesis among different insect orders due to different reproductive traits (Roy et al., 2018). In the migratory locust, *Locusta migratoria*, JH works through its receptor Methoprene-tolerant (Met) and Taiman (Tai) complex (Met-Tai) to mediate vitellogenesis and oocyte maturation (Song et al., 2014). In the firebug, *Pyrrhocoris apterus*, JH is synthesized after long-day condition stimulation to promote oogenesis through the Met-Tai complex (Smykal et al., 2014). Moreover, in dipterans, JH regulates the developmental stages in preparation for subsequent vitellogenesis and egg development rather than directly stimulating vitellogenesis (Raikhel et al., 2005) while 20E stimulates *Vg* expression and oocyte maturation after a blood meal in *Ae. Aegypti* (Raikhel et al., 2002). In many lepidopterans, including the tobacco hornworm, *Manduca sexta*, JH plays a vital role in vitellogenesis as Vg is synthesized in the adult stage, while in the silkworm, *Bombyx mori*, Vg is synthesized before adult ecdysis, and 20E is mainly responsible for vitellogenesis (Swevers and Iatrou, 2003; Telfer, 2009). In the coleopteran, *T. castaneum*, JH stimulates Vg production through the insulin-like peptide (ILP) signaling pathway (Parthasarathy et al., 2010b; Sheng et al., 2011) while 20E participates in ovarian growth and primary oocyte maturation (Parthasarathy et al., 2010a).

Nutrients are essential for insect reproduction. In anautogenous insects, such as mosquitoes, only after the absorption of proteins and amino acids (AAs) can vitellogenesis start (Attardo et al., 2005). For most insects, the insulin/insulin-like signaling (IIS) pathway and the AA/target of rapamycin (TOR) pathway are responsible for reproduction (Hansen et al., 2004; Abrisqueta et al., 2014). In *Ae. Aegypti* and the brown planthopper, *Nilaparvata lugens*, the silencing of *TOR* impedes the expression of *Vg* and reduces fecundity (Hansen et al., 2004; Lu et al., 2016). The inhibition of the expression of *ribosomal protein S6 kinase* (*S6K*), a downstream target of the TOR pathway, disrupts *Vg* expression and egg maturation (Hansen et al., 2005). In contrast, the translational repressor 4E-binding protein (4EBP) inhibits *Vg* expression (Roy and Raikhel, 2012). Furthermore, protein kinase B (Akt), the main effector for insulin signaling, activates TOR signaling (Hyun, 2013). A tyrosine kinase transmembrane insulin receptor (InR) has been identified in various insects, and the knockdown of InR significantly decreases the expression of *Vg2* in *T. castaneum* (Parthasarathy and Palli, 2011). Also, RNA interference (RNAi)-mediated InR knockdown reduces the fat body Vg messenger RNA (mRNA) level and oocyte growth in the German cockroach, *Blattella germanica* (Abrisqueta et al., 2014). In *T. castaneum* and the American cockroach, *Periplaneta americana*, TOR and ILP signaling could stimulate *Vg* expression and oocyte maturation (Parthasarathy and Palli, 2011; Zhu et al., 2020) and in dipterans, IIS/TOR signaling could stimulate ovarian ecdysteroidogenesis and mediate the secretion of 20E for Vg synthesis (Brown et al., 2009).

Although JH and 20E are the major regulators of insect reproduction, neuropeptides, like ILPs and ovary ecdysteroidogenic hormone, are also involved in the reproductive process that stimulates the ovaries of *Ae. Aegypti* to produce the steroid hormone ecdysone (Dhara et al., 2013). Meanwhile, a previous report shows that r-bursicon is able to stimulate Vg expression in the black tiger shrimp (Sathapondecha et al., 2015), the molecular mechanism remains unknown. In this study, we used *T. castaneum* as a model organism to study the impact of burs on insect reproduction and the underlying mechanism using RNAi and recombinant pburs (r-pburs). Our results indicate that burs influence the reproductive process through its receptor Tcrk to mediate the expression of *InR, Akt, TOR, S6K*, and *4EBP* and that the double-stranded RNA (dsRNA) effect can be passed from one generation to the next.

## Materials and Methods

### Insect Rearing and Staging

The *T. castaneum* Georgia-1 (GA-1) strain was reared on organic wheat flour containing 10% yeast at 28 ± 1°C under standard conditions (Parthasarathy et al., 2008). The pupae were distinguished by sex according to the structural differences in the genital papillae (Parthasarathy et al., 2008). Adults were staged as soon as they emerged, and adults with an untanned cuticle were staged as 0 h after emergence. Female and male adults were kept separately under the same conditions as described earlier.

### Double-Stranded RNA Synthesis and RNAi

The templates for dsRNA (dsGFP, dsburs, dspburs, and dsTcrk) synthesis were obtained by PCR to amplify the fragments of each gene using gene-specific primers containing the T7 polymerase promotor sequence at their 5′ ends (Supplementary Table 1), and the resulting complementary DNA (cDNA) was used as the template. dsRNA was synthesized using the purified PCR product and the HiScribe™ T7 Quick High Yield RNA Synthesis kit (E2050, New England Biolabs, Inc., Ipswich, MA, USA) following the methods described in the instruction manual. Synthesized dsRNA was purified using a phenol/chloroform extraction and isopropanol precipitation and dissolved in diethylpyrocarbonate-treated water (Tan and Palli, 2008). The concentration of dsRNA was measured using a Nanodrop 2000 spectrophotometer (Thermo Fisher Scientific, Inc., Wilmington, DE, USA) at 260 nm. dsRNA was injected into 2-day pupae on the ventral side between the first and second abdominal segment using the Nanoject II Auto-Nanoliter Injector (Drummond Scientific Co., Broomall, PA, USA) fitted with a 3.5-inch glass capillary tube pulled by a needle puller (Model P-2000, Sutter Instruments Co., Novato, CA, USA). In the preliminary dose-response experiment, 200 ng was found to be the optimum dose for burs, pburs, and Tcrk RNAi (data not shown), so each pupa was injected with 200 ng dsRNA in 50 nl (4 ng/nl). dsGFP was used as a control. The injected pupae were reared under standard conditions until use.

### Mating Assays

Double-stranded RNA-treated females 7-day post-emergence, in which the ovary is mature, were mated individually with the untreated virgin males in separate tubes (24 × 62 mm, 15 ml) containing wheat flour and yeast. A pair of mating beetles were placed in a tube and removed to new tubes every 24 h, allowing for egg deposition. Female fecundity was determined as the number of eggs laid per female per day. The hatch rate was calculated as the percentage of the number of hatched larvae vs. the total number of eggs laid.

### RNA Extraction, cDNA Synthesis, and Quantitative Real-Time PCR

Total RNA was extracted from the whole body of the staged beetles using the TRIzol reagent (Thermo Fisher Scientific, Inc., Waltham, MA, USA). DNA was eliminated from the total RNA using DNase I (Thermo Fisher Scientific, Inc., Waltham, MA, USA), and 1 μg of total RNA from each sample was used for cDNA synthesis in a 20 μl reaction volume using a High-Capacity cDNA Reverse Transcription kit (Thermo Fisher Scientific, Inc., Waltham, MA, USA) with a RNase inhibitor following the instruction of the manufacturer. Quantitative real-time PCR (qRT-PCR) was performed using the QuantStudio 3 Real-Time PCR System (Thermo Fisher Scientific, Inc., Waltham, MA, USA). qRT-PCR reaction components were: 1 μl of cDNA (100 ng/μl), 1 μl each of forward and reverse sequence specific primers (10 pmol/μl) (Supplementary Table 1), 3 μl of ddH_2_O, and 5 μl of iTaq™ Universal SYBR^®^ Green Supermix (Biorad Laboratories, Hercules, CA, USA). qRT-PCR programs were 95°C for 3 min, 45 cycles of 95°C for 10 s, 60°C for 20 s, 72°C for 30 s, and 65–95°C at 0.5°C increments for 2–5 s. The relative levels of mRNAs were quantified three times and normalized using an internal control (*T. castaneum* ribosomal protein S3, Tcrp3) (Lord et al., 2010). The primer sequences for the target genes are listed in Supplementary Table 1. The relative expression levels of genes were calculated according to the 2^−ΔΔCT^ method (Livak and Schmittgen, 2001). Three biological replicates were performed to measure mRNA levels by qRT-PCR.

### Vg Protein Determination

Six complete ovaries dissected from 8-day females or 50 eggs laid by different RNAi-treated mating groups were used as a biological replicate. Three biological replicates were used for ovarian or egg samples. Each ovarian or egg sample was homogenized with 100 μl phosphate-buffered saline (PBS) (137 mM NaCl, 2.7 mM KCl, 10 mM Na_2_HPO_4_, and 1.8 mM KH_2_PO_4_, pH 7.4). Protein (20 μg) from each sample was denatured at 95°C for 5 min in 4× loading buffer (0.2 M Tris-HCl, 0.4 M dithiothreitol (DTT), 8.0% (w/v) sodium dodecyl sulfate (SDS), 6 mM bromophenol blue, and 40% glycerol). Denatured protein samples were separated by 8% reducing SDS-polyacrylamide gel electrophoresis (SDS-PAGE) and stained with Coomassie Brilliant Blue R-250. The size of Vg bands was determined according to molecular weight (MW) standards (PageRuler™ Plus Prestained Protein Ladder, 10–250 kDa, Thermo Fisher Scientific, Inc., Waltham, MA, USA). The hemolymph from male adults was used as a negative control, and the protein sample from 1-day eggs was used as a positive control for the determination of Vg. The Vg protein bands were analyzed and quantified by GeneTools from Syngene (Cambridge, UK).

### Ovary, Egg, and Beetle Phenotypes After RNAi

Female adults 7–9-days post-emergence were separately collected after dsburs, dspburs, or dsGFP injection. The ovaries and ovarioles from dsRNA-treated and control groups were dissected, placed in Ringer's solution (130 mM NaCl, 4 mM KCl, 3 mM CaCl_2_, and 12 mM NaHCO_3_, pH 7.4), and photographed using a Leica M205 C stereomicroscope with a digital camera (Leica Microsystems, Wetzlar, Germany). The offspring eggs, larvae, pupae, and adults of different dsRNA-treated groups were also photographed as described earlier.

### Cloning, Expression, and Purification of r-pburs Protein

The cDNA encoding the mature peptide of pburs was amplified using the corresponding primers listed in Supplementary Table 1. The fragment was then ligated into pET-32a (+) plasmids with His-tag using the In-Fusion^®^ HD Cloning Plus kit (Takara Bio Inc., San Jose, CA, USA) following the instruction of the manufacturer, and transformed into *Escherichia coli* BL21 (DE3) cells for protein expression. r-pburs protein was expressed in a soluble form and purified with Pierce™ Ni-NTA Magnetic Agarose (Thermo Fisher Scientific, Inc., Carlsbad, CA, USA) according to the protocol of the manufacturer. Because burs protein could not be expressed in a soluble form, it was not used for purification and subsequent assays. The purified r-pburs protein was concentrated and desalted using an Amicon^®^ Ultra-4 Centrifugal Filter Unit (Millipore Sigma, Cork, Ireland). r-pburs protein was separated by 12% SDS-PAGE and visualized after Coomassie Brilliant Blue R-250 staining for its purity determination.

### Western Blot and r-pburs Protein Injection

The purified r-pburs protein was separated by 12% SDS-PAGE under non-reducing and reducing conditions and then transferred to a nitrocellulose (NC) membrane (0.2 μm). After protein transfer, the membrane was blocked in 10 ml of TBS (20 mM Tris, 150 mM NaCl, pH 7.4) containing 0.05% Tween-20 (TBST) and 5% non-fat milk for 1 h at room temperature. Following three washes with TBST, the membrane was incubated with a 6x-his tag monoclonal antibody (Thermo Fisher Scientific, Inc., Waltham, MA, USA) at a 1:2,000 dilution in 5% bovine serum albumin (BSA) overnight at 4°C. After incubation, the membrane was washed three times with TBST and incubated with a goat anti-mouse IgG (H + L) horseradish peroxidase (HRP) conjugate (Thermo Fisher Scientific, Inc., Waltham, MA, USA) diluted 1:5,000 in TBST at room temperature for 1 h. The r-pburs immunosignal was developed in a SuperSignal™ West Pico PLUS Chemiluminescent Substrate Solution (Thermo Fisher Scientific, Inc., Waltham, MA, USA) according to the instructions of the manufacturer and detected using G:Box (Syngene, Cambridge, UK).

To investigate the role of pburs in reproduction, r-pburs (50 ng protein in 120 nl) or a blank-vector transfected sample (control, the identical volume purified with Pierce™ Ni-NTA Magnetic Agarose as described earlier for r-pburs purification) was injected into 1-day virgin females on the ventral side between the first and second abdominal segment using the same method as described earlier for dsRNA injection. The female adults 3, 6, 12, 24, 48, and 72 h post r-pburs injection were collected for RNA extraction and qRT-PCR detection. For the rescue assay, r-pburs was injected 3 days post-dsRNA treatments in 2-day female pupae. After 72 h, the females were collected for RNA extraction and qRT-PCR detection.

### Statistical Analysis

The mean values of gene expression levels and other parameters between RNAi-treated and control groups were compared by Student's *t*-test and ANOVA using the graphic software Prism (Graph Pad Software, v8.1.2, San Diego, CA, USA). All data obtained were presented as the mean ± SEM from three or more independent experiments. The value of *p* < 0.05 was regarded as statistically significant.

## Results

### Developmental Expression Profiles of *burs, pburs, Tcrk, Vg1, Vg2*, and *VgR*

**Figure 1:**
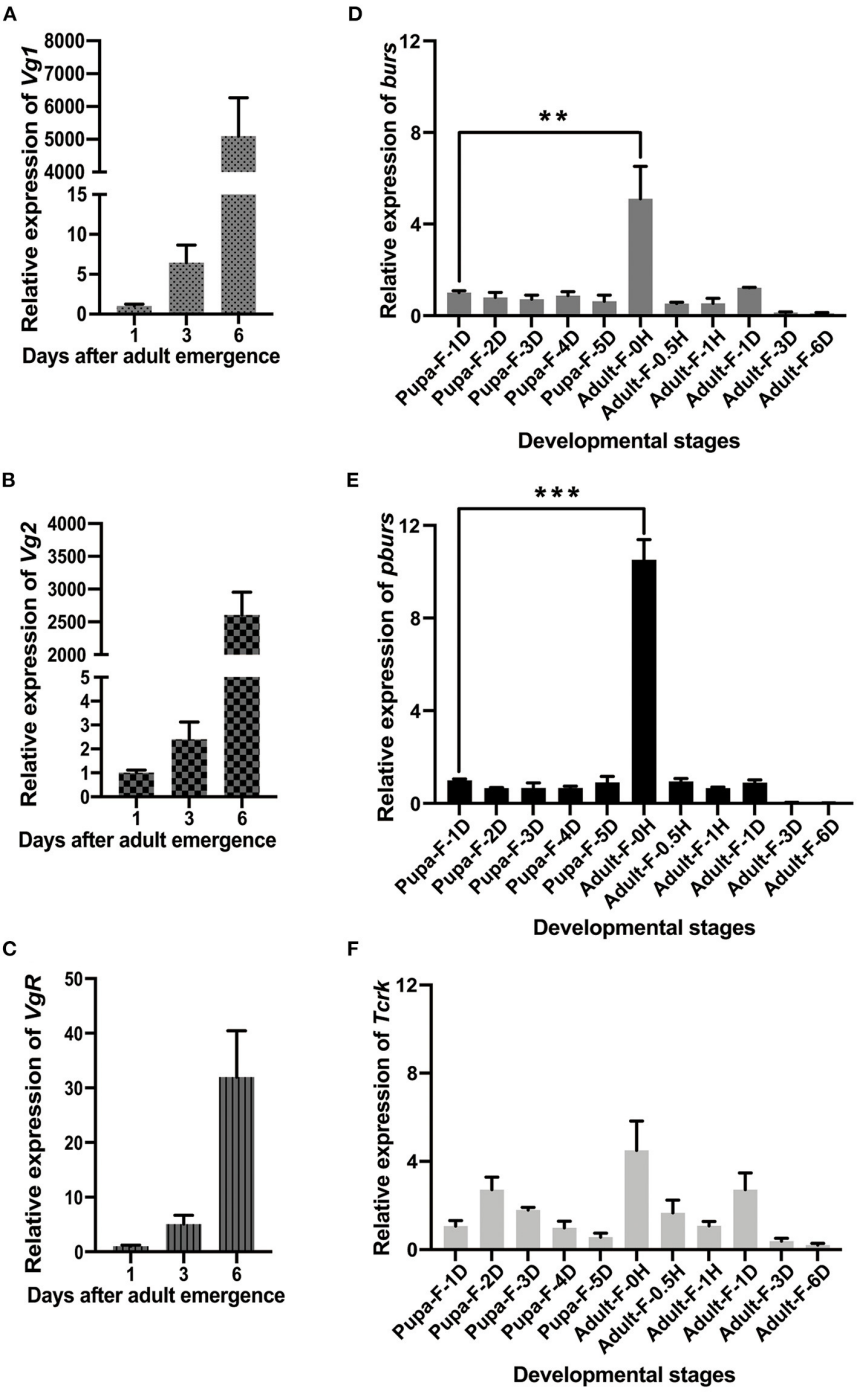
Developmental expression profiles of *Vg1, Vg2, Vg receptor* (*VgR*), *bursicon* (*burs*), *partner of burs* (*pburs*), and *buricon receptor T. castaneum rickets (Tcrk)*. **(A–C)** Relative expression levels of *Vg1, Vg2*, and *VgR* in 1-, 3-, and 6-day females. **(D–F)** Relative expression levels of *burs, pburs*, and *Tcrk* from the pupal to the adult stage. The bars represent the mean ± SEM. The asterisks above bars indicate significant differences between the treatment and the corresponding control, ^**^p < 0.01, ^***^p < 0.001 by *t-*test.

Quantitative real-time PCR analyses revealed that the expression levels of *Vg1, Vg2*, and *VgR* showed an upward trend in 1–6-day female adults with a dramatic increase on day 6 (Figures 1A–C) while *burs* and *pburs*, which were mainly expressed in the central nervous system (Supplementary Figures 1A,B), were expressed throughout all developmental stages, with distinguished prominent peaks in the newly emerged female adults (Figures 1D,E). The expression peaks of burs and pburs in the newly emerged females were prior to the expression peaks of *Vg1, Vg2*, and *VgR* (Figures 1A–C). Although *Tcrk* was also mainly expressed in the central nervous system (Supplementary Figure 1C), the developmental expression profile indicated that it is constitutively expressed (Figure 1F).

### Effects of burs, pburs, and Tcrk RNAi on the Expression of *Vg1, Vg2*, and *VgR*

**Figure 2:**
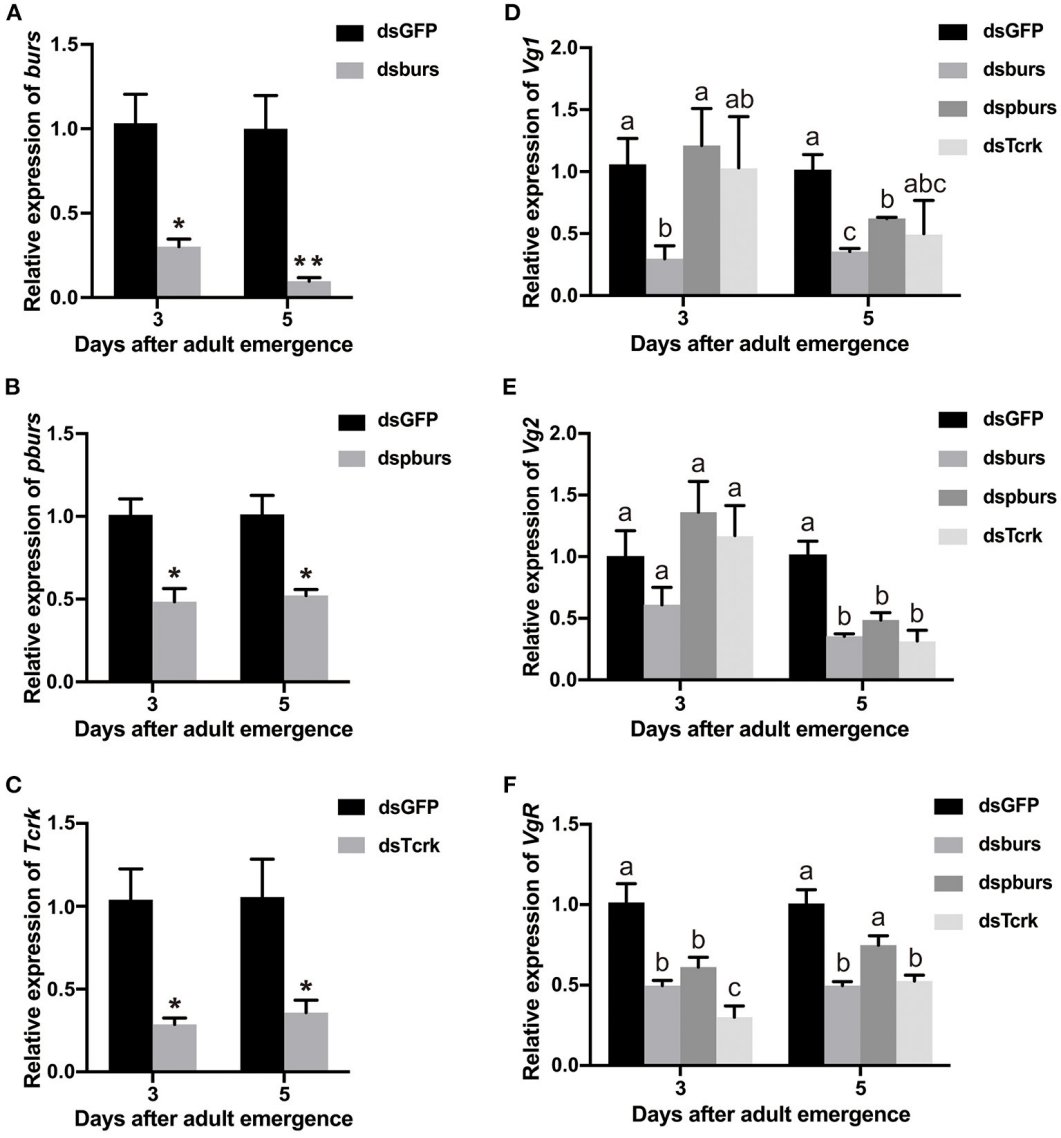
The efficiency of burs, pburs, or Tcrk RNA interference (RNAi) and their effects on the expression of *Vg1, Vg2*, and *VgR*. **(A–C)** Relative expression levels of *burs, pbur*, or *Tcrk* in 3- and 5-day females after dsGFP, dsburs, dspburs, or dsTcrk treatments. **(D–F)** Relative expression levels of *Vg1, Vg2, and VgR* in 3- and 5-day females after dsGFP, dsburs, dspburs, or dsTcrk treatments. The asterisks above bars indicate significant differences between the treatment and the corresponding control, ^*^p < 0.05, ^**^p < 0.01 by *t*-test. Different letters on the bars indicate the means ± SEM, which are significantly different (*p* < 0.05) among treatments by ANOVA.

After the injection of dsburs, dspburs, or dsTcrk into 2-day female pupae, the expression levels of *burs, pburs*, or *Tcrk* were significantly decreased in 3- and 5-day adults (Figures 2A–C). The efficiency of burs RNAi reached 60% in 3-day adults and 80% in 5-day adults (Figure 2A), whereas the efficiency of pburs RNAi was about 50% in the same condition (Figure 2B). The silencing efficiency of *Tcrk* was about 70 and 65% in 3- and 5-day females, respectively (Figure 2C). The silencing of burs significantly downregulated the expression levels of *Vg1* and *VgR* in 3- and 5-day adults and inhibited the expression of *Vg2* in 5-day adults (Figures 2D–F). After pburs RNAi, the expression levels of *Vg1* and *Vg2* were suppressed in 5-day adults while the mRNA level of *VgR* was downregulated in 3-day adults (Figures 2D–F). The knockdown of *Tcrk* downregulated the expression level of *VgR* by about 70 and 50% in 3- and 5-day females, respectively (Figure 2F), and *Vg2* by about 70% in 5-day females (Figure 2E) but had no significant effect on the expression level of *Vg1* (Figure 2D).

### Phenotypes of the Ovary and Ovariole After burs or pburs RNAi

**Figure 3:**
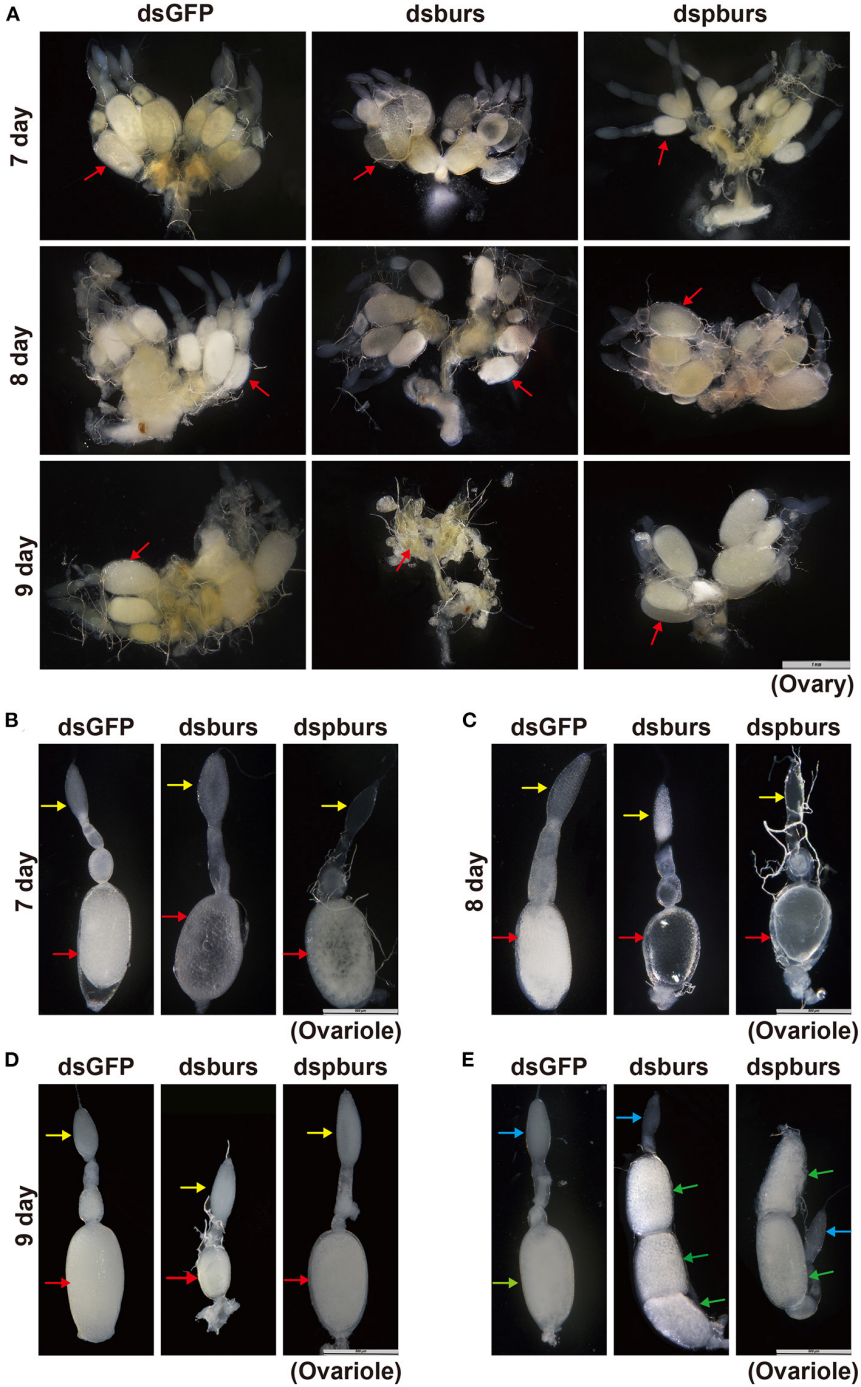
Phenotypes of the ovary and ovariole in burs or pburs RNAi beetles. **(A)** The ovaries from 7-, 8-, and 9-day females after dsGFP, dsburs, and dspburs treatments. **(B–D)** The ovarioles from 7-, 8-, and 9-day females after dsGFP, dsburs, and dspburs treatments. **(E)** The ovarioles from the mated females 7-day post-dsGFP, dsburs, and dspburs treatments. The red arrows indicate oocytes from the unmated females, and the yellow arrows indicate germarium from the unmated females. The green arrows indicate oocytes from the mated females, and the blue arrows indicate germarium from the mated females.

To further investigate *in vivo* function of burs and pburs and their potential roles in ovarian development, we observed the morphology of ovaries and ovarioles isolated from RNAi beetles at various time points after adult eclosion. The ovaries of *T. castaneum* contain approximately eight telotrophic ovarioles (Bai and Palli, 2016). Each ovariole comprises two distinct structures: the germarium that contains nurse cells and developing follicle cells and the vitellarium that includes the primary oocytes surrounded by follicle cells. Our data showed that there was no significant difference in the number and size of the ovary, ovariole, and oocyte in 7- and 8-day female adults between burs- or pburs-silenced and control groups, but the oocyte in these groups was much transparent than that in control (Figures 3A–C and Supplementary Figures 2A–F). However, the ovaries, ovarioles, and oocytes of 9-day females in the burs RNAi group were much smaller than the ovaries, ovarioles, and oocytes of 9-day females in the control group (Figures 3A,D and Supplementary Figures 2A–C,E,F). Interestingly, most mated 7-day females post burs or pburs RNAi had 2–3 full-size oocytes in each ovariole, instead of one presented in the control group (Figure 3E), suggesting that the immature oocytes were retained in the ovariole after RNAi, possibly due to the lack of Vg content.

Subsequently, we tested this hypothesis by quantifying Vg content in dsburs- or dspburs-treated and dsGFP control ovaries. The results showed that the silencing of burs or pburs significantly reduced Vg content in 8-day ovaries compared with control ovaries (Supplementary Figure 3), and similar results were also obtained in 7- and 9-day ovaries (data not shown).

### Effects of burs and pburs RNAi on the Fecundity, Hatch Rate, Vg Content, and Embryonic Development

**Figure 4:**
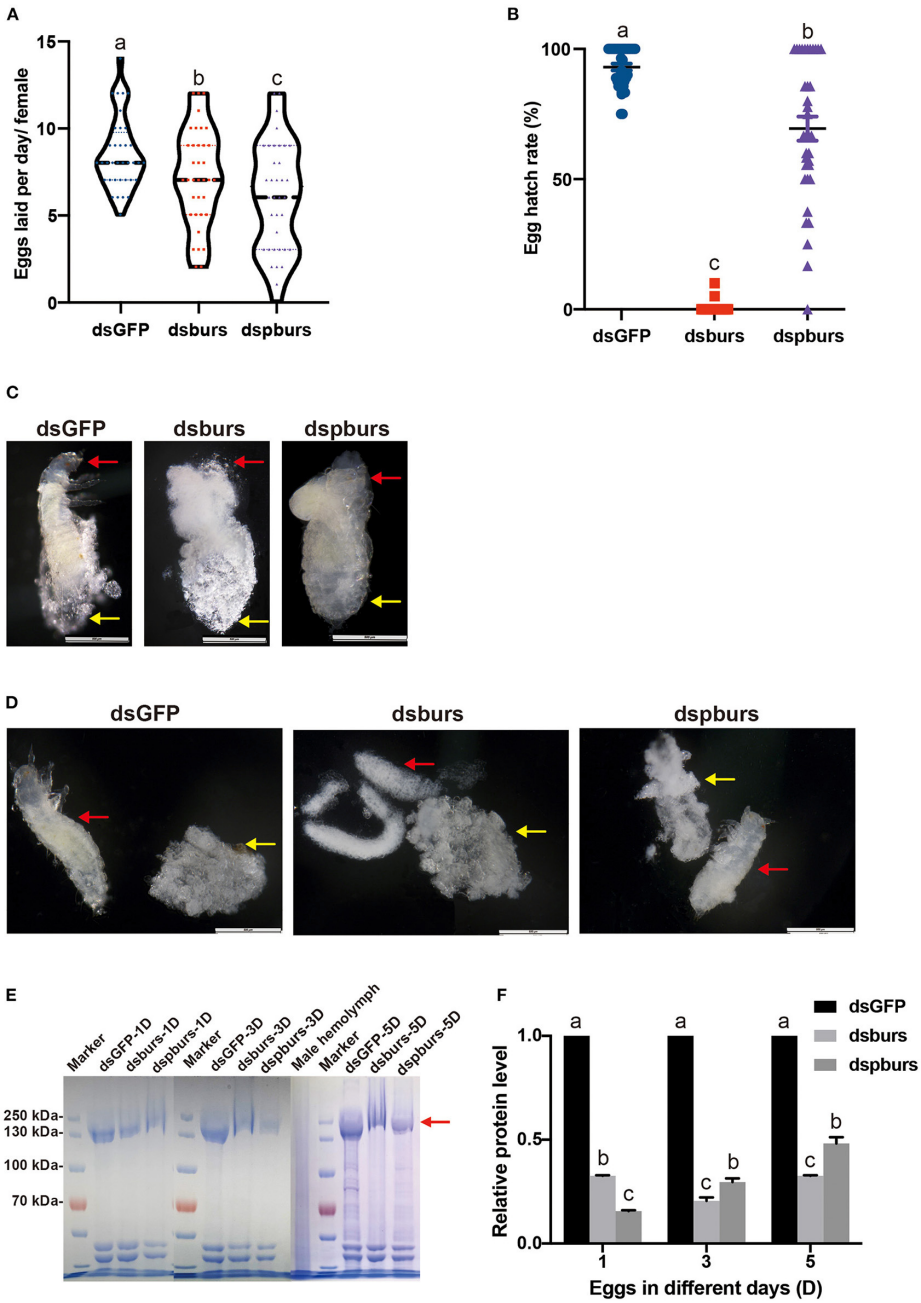
Effects of dsburs and dspburs treatments on egg production, hatch rate, morphology, and protein content. **(A)** The number of eggs laid per day per female in dsGFP-, dsburs-, and dspburs-treated beetles. **(B)** Egg hatch rate in dsGFP-, dsburs-, and dspburs-treated females after mating. **(C)** The morphology of 5-day eggs from dsGFP-, dsburs-, and dspburs-treated groups. **(D)** Eggshells and immature larvae from 5-day eggs from dsGFP-, dsburs-, and dspburs-treated groups. The red arrows indicate the larvae, and the yellow arrows indicate the eggshells. **(E)** Protein in 1-, 3-, and 5-day eggs from dsGFP-, dsburs-, and dspburs-treated groups. **(F)** The quantification of vitellogenin (Vg) protein bands in the Coomassie Brilliant Blue-stained gel **(E)**. The bars represent the means ± SEM. Different letters on the bars indicate the means ± SEM, which are significantly different (*p* < 0.05) among treatments by ANOVA.

After burs or pburs silencing in 2-day female pupae, 7-day female adults with mature ovaries were allowed to mate with the untreated males for 24 h in a tube, then the pair was removed from the tube to a new tube with diet. Six mating pairs were used, and eggs were counted daily for 6 days. We sieved eggs and counted the number of eggs using a dissecting microscope. To evaluate the hatch rate and larval development ability, we returned eggs and flour to their tubes and held them under the same conditions as described earlier. After 2 weeks, the number of larvae was counted and compared with the number of eggs for the hatch rate calculation. Compared with the average of 8.5 eggs laid per female per day in the dsGFP control group, the average number of eggs laid in the dsburs-treated group was 7.2, down by 15.3% (Figure 4A). Meanwhile, the fecundity of females in the dspburs-treated group was 6.1, down by 28.2% (Figure 4A). The egg hatching rate in dsburs-treated females was close to zero, indicating that there was almost no successful hatching (Figure 4B). The egg hatch rate in the dspburs-treated group varied from 0 to 100%, with an average of 69.41%, which was significantly lower than the average hatch rate of 93.07% in the dsGFP-treated group (Figure 4B).

To investigate why eggs laid by dsburs-treated beetles failed to hatch successfully, we dissected 5-day eggs, which were about to hatch at the standard development time. It was apparent that the 5-day eggs of control and pburs RNAi groups contained a complete larval body after stripping off the eggshells, while the eggs from dsburs-treated females did not have a larval shape, which might be the reason for the failed hatch (Figures 4C,D).

We also assayed the Vg protein content in the eggs from dsburs- or dspburs-treated females. The Vg protein content from the eggs laid by dsburs- and dspburs-treated groups were much lower than the Vg protein content from the eggs laid by the dsGFP control group (Figures 4E,F), suggesting that burs and pburs RNAi reduced *Vg* expression in females, leading to decreased Vg content in the eggs.

### Impact of pburs RNAi on Offspring

**Figure 5:**
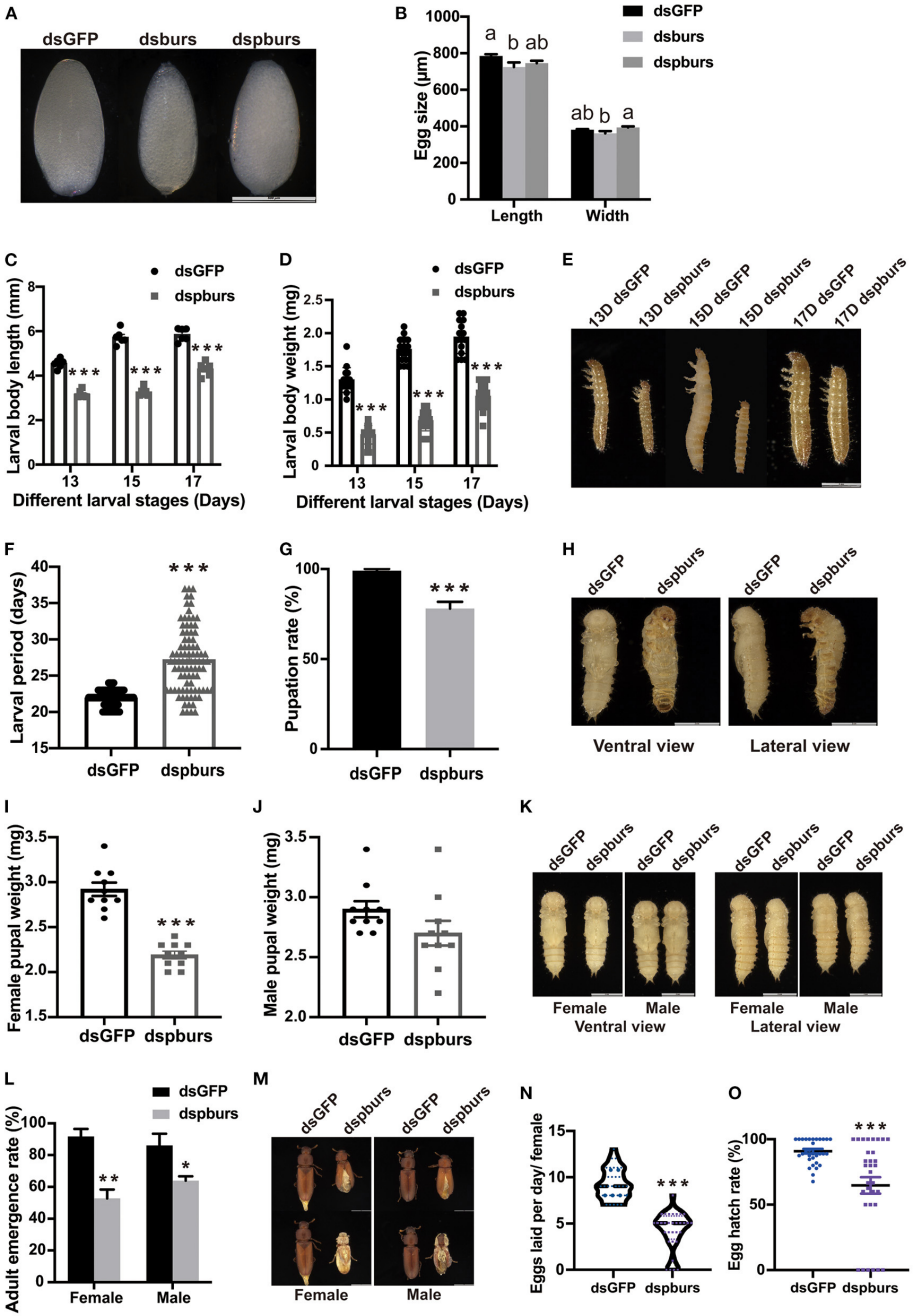
Impacts of pburs RNAi on offspring growth and development. **(A)** Morphology and **(B)** length and width of eggs from dsGFP-, dsburs-, and dspburs-treated groups. **(C)** Body length, **(D)** body weight, and **(E)** morphology of 13-, 15-, and 17-day F1 larvae from dsGFP- and dspburs-treated groups. **(F)** F1 larval developmental periods in dsGFP- and dspburs-treated groups. **(G)** Pupation rate in dsGFP- and dspburs-treated groups. **(H)** Ventral and lateral view of pupae from dsGFP- and dspburs-treated groups. **(I)** Bodyweight of female pupae and **(J)** bodyweight of male pupae from dsGFP- and dspburs-treated groups. **(K)** F1 female and male pupae in dsGFP- and dspburs-treated groups. **(L)** Adult emergence rate in F1 female and male beetles in dsGFP- and dspburs-treated groups. **(M)** Phenotypes of F1 female and male beetles in dsGFP- and dspburs-treated groups. **(N)** Egg production per day per mated female in dsGFP- and dspburs-treated F1 beetles. **(O)** Egg hatch rate of dsGFP- and dspburs-treated mated F1 females. Different letters on the bars indicate the means ± SEM, which are significantly different (*p* < 0.05) among treatments by ANOVA. Asterisks above bars indicate significant differences between the treatment and corresponding control, ^*^p < 0.05, ^**^p < 0.01, ^***^p < 0.001 by *t-*test.

For the egg size measurement, we dissected the eggs from the ovaries of the female beetles that had mated for 1 day after dsRNA treatment. Compared with the control group, the eggs from the dsburs-treated group were significantly smaller in length while there was no difference in the length and width of eggs between the dspburs-treated group and the dsGFP control (Figures 5A,B).

For the next generation of larvae, we chose 13-, 15-, and 17-day larvae to evaluate the effects of dspburs on offspring development Because the eggs produced by burs RNAi females failed to hatch, we focused on the influence of silencing pburs in parental beetles on the offspring larval size, larval developmental period, pupation rate, and other parameters. Compared to the larvae in the control group with the average length of 4.552, 5.727, and 5.850 mm, respectively, in the three periods, the larvae from the dspburs-treated group averaged only 3.17, 3.27, and 4.30 mm, down by 30.4, 42.9, and 26.5%, respectively (Figure 5C). In addition to the differences in body length, the offspring larvae from the dspburs-treated group also had a significant weight reduction, with the average weight being ~50% that of the control group over these three periods (Figure 5D). The corresponding phenotypes are shown in Figure 5E. Furthermore, the offspring larval developmental duration was significantly extended in the dspburs-treated group with an average of 27.2 days, compared to 21.9 days in the control group (Figure 5F), showing a delay of 24.2%. Additionally, control larvae were able to pupate successfully at 99% while only 78% of the larvae from the dspburs-treated group were able to pupate (Figure 5G), and a noticeably different phenotype was also observed in the dspburs-treated cohort (Figure 5H).

For the offspring pupae, we measured the weight of 2-day female and male pupae. The average weight of female offspring pupae in the dspburs-treated group was 2.19 mg, compared with 2.92 mg in the dsGFP-treated group, down by 25% (Figure 5I). However, there was no significant difference in the weight of male pupae between dspburs-treated and control groups (Figure 5J). The differences in body weight between the female and male pupae after successful pupation in the different treatments are shown in Figure 5K.

In addition to these effects, dspburs treatment also impacted the offspring's adult eclosion rate. Compared to the control with 91.67 and 86.11% eclosion rate in females and males, respectively, only 52.78% of the females and 63.89% of the males in the dspburs-treated group were able to eclose successfully (Figure 5L). The phenotype of abnormal eclosion (stocky body with split elyte) is shown in Figure 5M. After offspring adults were paired and mated, we measured the fecundity and hatch rate as described previously. The average number of eggs laid by the offspring adults from the dspburs-treated group was 4.2 per female per day, down by 54.3%, compared with 9.2 per female per day in the control group (Figure 5N). The egg hatching rate from the dspburs-treated group was 64.71%, down by 26.13% compared to the control group (Figure 5O).

Because the silencing of pburs had a significant impact on offspring growth and development, we turned to measure the expression of *pburs, Vg1, Vg2*, and *VgR* in the 3-day offspring larvae and female adults to investigate the long-term effect of dsRNA on offspring. The 15-day offspring larvae from the dspburs-treated group had a significantly lower expression level of *pburs* than that of the control group (Supplementary Figure 4A). The expression levels of *pburs* and *Vg2* of the 3-day offspring female adults from the pburs RNAi group were also significantly reduced compared with the control group (Supplementary Figures 4A–D), suggesting that pburs RNAi has a long-lasting effect on the offspring.

### Effects of burs and pburs RNAi on JH and Insulin/Insulin-Like/TOR Signaling

**Figure 6:**
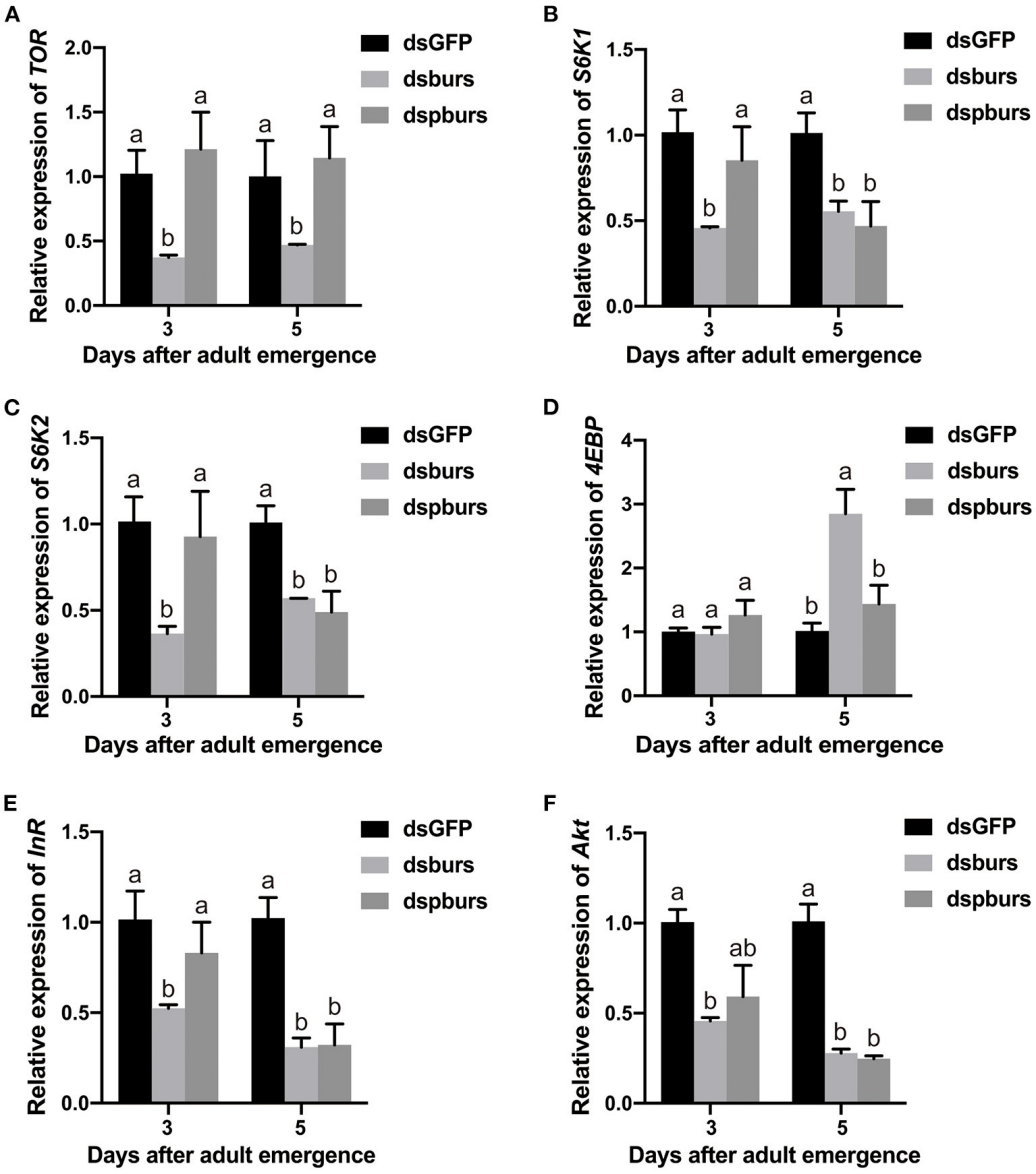
Effects of burs and pburs RNAi on the insulin/insulin-like signaling (IIS)/target of rapamycin (TOR) pathway. **(A–D)** Relative expression levels of the TOR pathway genes *TOR, S6K1, S6K2*, and *4EBP* in 3- and 5-day female adults after the silencing of burs or pburs. **(E,F)** Relative expression levels of the IIS pathway genes insulin receptor (*InR*) and protein kinase B (*Akt*) in 3- and 5-day female adults after burs or pburs silencing. Different letters on the bars indicate the means ± SEM, which are significantly different (*p* < 0.05) among treatments by ANOVA.

As the silencing of burs or pburs affected reproduction, we speculated that burs homodimers might influence the IIS/TOR pathway. To test this hypothesis, we first knocked down burs or pburs *via* RNAi and then measured the expression levels of the genes involved in a pathway. Our results indicated that burs silencing significantly inhibited the expression levels of *TOR, S6K1*, and *S6K2* by ~50% in 3- or 5-day female adults (Figures 6A–C) while the expression of the TOR inhibitor *4EBP* increased significantly after burs RNAi in 5-day adults (Figure 6D). After silencing pburs, the expression of *S6K1* and *S6K2* was significantly reduced in 5-day females (Figures 6B,C). For the IIS pathway, the expression levels of the receptor gene *InR* and the main effector gene *Akt* were significantly downregulated in 3- and/or 5-day females after burs or pburs RNAi (Figures 6E,F). We also investigated the impact of burs or pburs RNAi on the genes related to JH synthesis (*JHAMT*), JH receptors (*Met* and *Tai*), and JH esterase (*JHE*). The results showed that burs or pburs RNAi had no influence on the expression of *JHAMT, Met*, and *JHE* (Supplementary Figures 5A,B,D), but did increase *Tai* expression (Supplementary Figure 5C). These results suggest that burs and pburs may regulate reproduction by influencing JH and IIS/TOR signaling pathways.

### Effects of r-pburs Protein on the Expression of *Vg* and IIS/TOR Pathway- and JH-Related Genes

**Figure 7:**
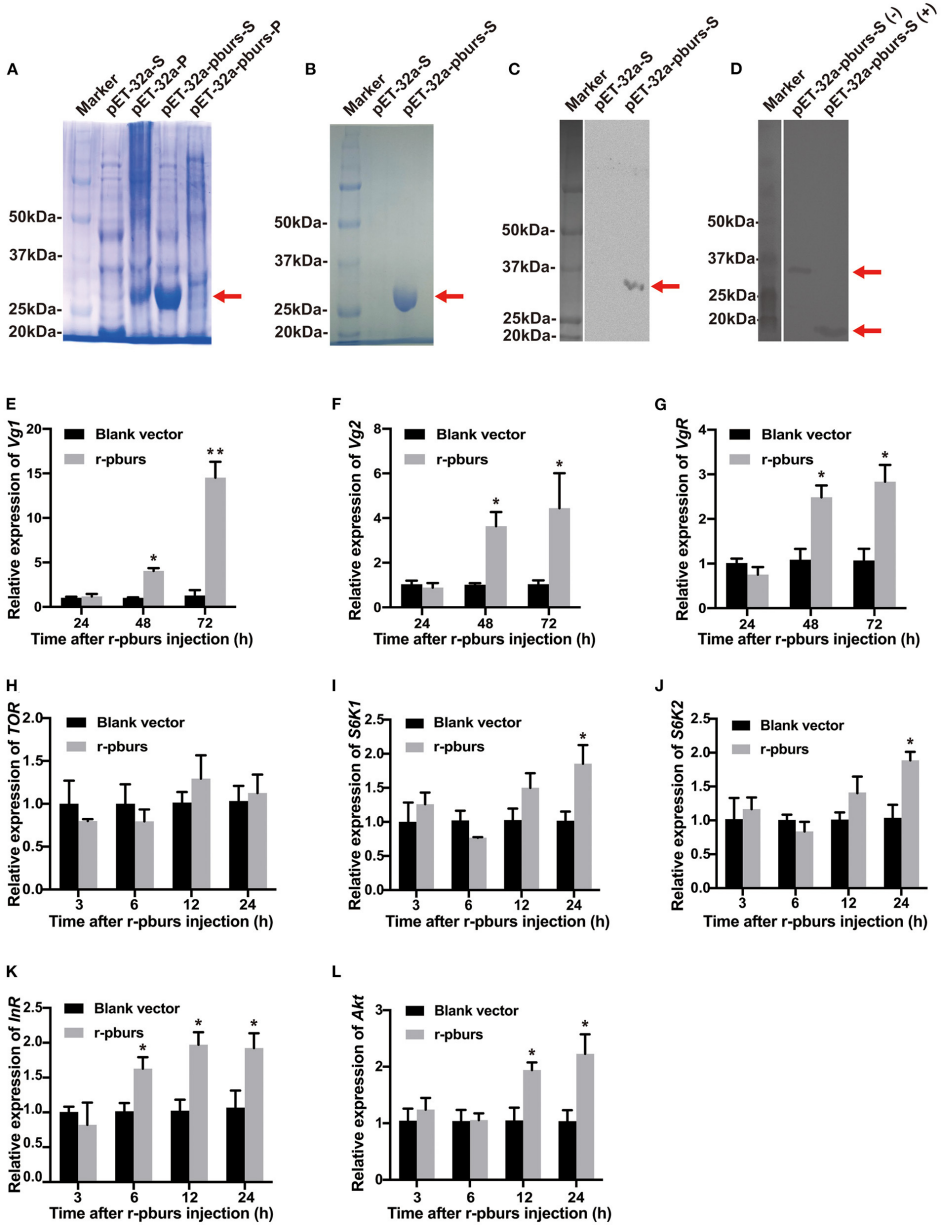
Effects of recombinant pburs (r-pburs) protein injection on the expression of *Vg* and IIS/TOR pathway genes. **(A)** Coomassie Blue staining of recombinant protein separated by sodium dodecyl sulfate-polyacrylamide gel electrophoresis (SDS-PAGE). Marker: the protein molecular weight (MW) marker; pET-32a-S: the supernatant protein of *E. coli* transfected with pET-32a; pET-32a-P: the precipitate of *E. coli* with pET-32a; pET-32a-pburs-S: the supernatant protein of *E. coli* with pET-32a-pburs; pET-32a-pburs-P: the precipitated protein of *E. coli* with pET-32a-pburs. **(B)** SDS-PAGE of the purified r-pburs protein. **(C)** Western blotting of control and r-pburs protein under a non-reducing condition. **(D)** Western blotting of the non-reduced (–) and reduced (+) r-pburs protein. Arrows indicate the location of r-pburs proteins. **(E–G)** Relative expression levels of *Vg1, Vg2*, and *VgR* in females 24, 48, and 72 h post-r-pburs injection into 1-day female adults. **(H–L)** Relative expression levels of *TOR, S6K1, S6K2, InR*, and *Akt* in females 3, 6, 12, and 24 h post r-pburs protein injection into 1-day female adults. The bars represent the means ± SEM. Asterisks above bars indicate significant differences between the treatment and corresponding control, ^*^p < 0.05, ^**^p < 0.01 by *t*-test.

As shown in Figure 7A, the only r-pburs protein was successfully expressed in the supernatant and precipitate. Consequently, r-pburs was used to evaluate its effect on Vg-related gene expression. Compared to the negative control [pET-32a (+) vector without a pburs insert], a distinct band of r-pburs with a molecular mass of ~32 kDa was detected and separated by SDS-PAGE (Figure 7A), Next, r-pburs was successfully purified from the supernatant using Ni-NTA Magnetic Agarose beads (Figure 7B) and verified using western blotting (Figure 7C). r-pburs protein under reducing and non-reducing conditions is shown in Figure 7D. These results indicate that r-pburs was successfully expressed *in vitro* and purified to near homogeneity.

We injected the purified r-pburs into 1-day females (when hemolymph *Vg* titer is low) using blank-vector transfected samples (purified using the same protocol for r-pburs purification) as a control. Female adults 3, 6, 12, 24, 48, and 72 h post-r-pburs injection were collected for RNA extraction and qRT-PCR analyses of Vg-related and IIS/TOR pathway genes. The expression of *Vg1, Vg2*, and *VgR* increased significantly (Figures 7E–G) 48 and 72 h post r-pburs injection. Although it had no influence on the TOR transcript level (Figure 7H), it significantly increased the expression of *S6K1* and *S6K2* 24 h post-r-pburs injection compared with the control (Figures 7I,J). The expression of *InR* was upregulated 6–24 h after r-pburs injection (Figure 7K) and the *Akt* mRNA level was increased significantly 12 and 24 h post r-pburs injection compared with the control (Figure 7L). Because the r-pburs-stimulated expression of *S6K1, S6K2, InR*, and *Akt* in the IIS/TOR signaling pathway is ahead of *Vg* and *VgR* expression, we speculate that pburs regulates *Vg* and *VgR* expression likely through the IIS/TOR signaling pathway.

Because JH has been shown to mediate the vitellogenesis process in *T. castaneum* (Parthasarathy et al., 2010b; Sheng et al., 2011), we went further to investigate the influence of r-pburs on the expression of JH-related genes in previtellogenic females. Compared to the control, the expression levels of both JH synthesis-related gene *JHAMT* and JH receptor genes, *Met* and *Tai*, increased significantly 6–24 h post r-pburs injection (Supplementary Figures 5E–G). However, r-pburs did not influence the mRNA level of *JHE* (Supplementary Figure 5H). Thus, the quick responses of JH synthesis-related and JH receptor genes to r-pburs injection suggest that pburs could also mediate vitellogenesis by triggering the JH signal in *T. castaneum*.

### Rescue Effects of r-pburs in the dspburs or dsTcrk-Treated Females

**Figure 8:**
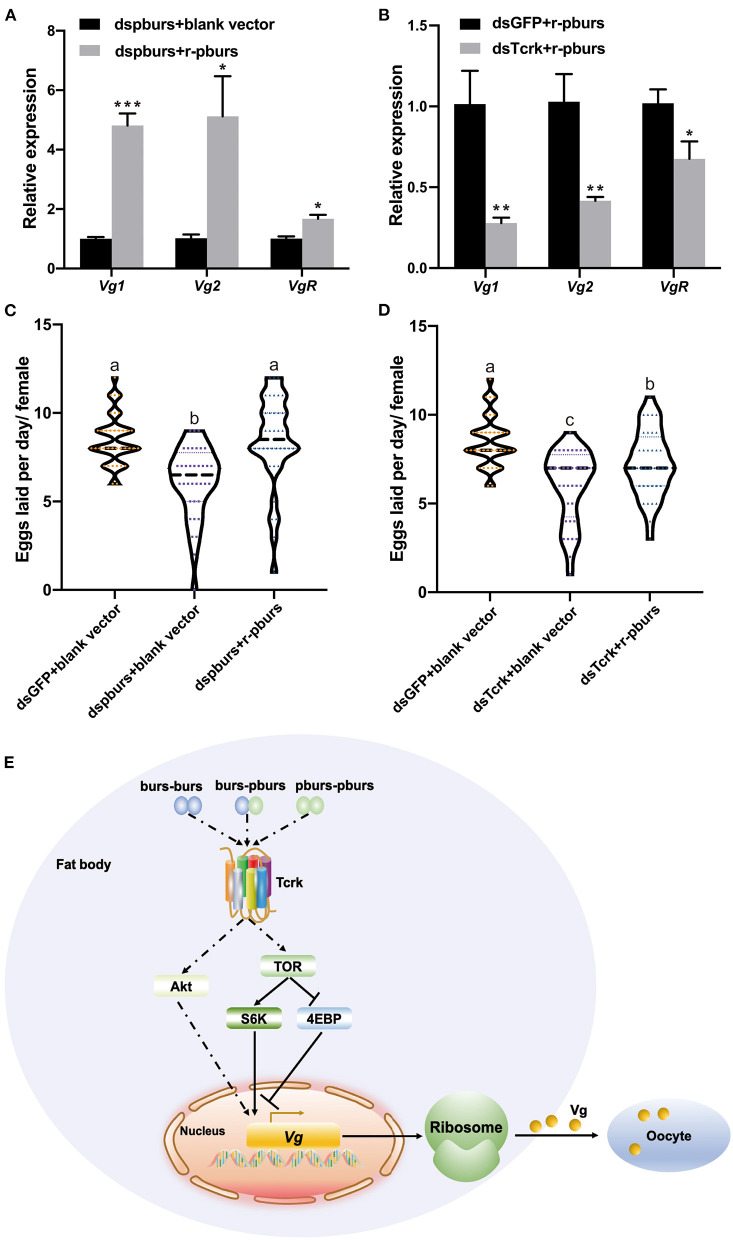
Effects of r-pburs protein on Vg-related genes and egg production in dspburs- or dsTcrk-treated females and a hypothetical model of bursicon (burs) signaling pathways related to *Vg* expression in *Tribolium castaneum*. The relative expression of *Vg1, Vg2*, and *VgR* in **(A)** dspburs- or **(B)** dsTcrk-treated females 72 h after r-pburs protein injection. The number of eggs laid per day per female in **(C)** dspburs- or **(D)** dsTcrk-treated beetles after r-pburs protein injection. The bars represent the means ± SEM. Asterisks above bars indicate significant differences between the treatment and corresponding control, ^*^p < 0.05, ^**^p < 0.01, ^***^p < 0.001 by *t*-test. Different letters on the bars indicate the means ± SEM, which are significantly different (*p* < 0.05) among treatments by ANOVA. **(E)** The hypothetical model shows the interactions between bursicon and key components in the Vg synthesis and uptake. The solid arrow indicates a direct activation signal, the dotted arrow indicates an indirect activation signal, and “⊥” indicates the suppression signal.

To further investigate if r-pburs could rescue reproduction in dspburs-treated females, we injected r-pburs into dspburs-treated females and assayed the expression levels of *Vg1, Vg2*, and *VgR* 3 days post-r-pburs injection. The results showed that r-pburs could increase the expression of these three genes in pburs-silenced females (Figure 8A). As expected, r-pburs failed to rescue, actually inhibited *Vg1, Vg2*, and *VgR* expression in *Tcrk* knockdown females (Figure 8B). We then went further to investigate the rescue effect on egg production. The number of eggs laid by dspburs-treated females was 8.4 per day after the r-pburs injection, increased by 37.46% compared to control (Figure 8C). These results indicate that r-pburs could fully rescue the inhibitory effect caused by dspburs treatment. However, r-pburs could not rescue egg production in dsTcrk-treated females (6 eggs per day in the dsTcrk-treated females vs. 8.5 eggs per day in the dsGFP-treated females) (Figure 8D). It must be noted that as shown in Figure 8D, the number of eggs laid by the dsTcrk-treated females was slightly but not fully rescued by r-pburs, presumably due to the action of residual Tcrk after RNAi as RNAi efficiency of Tcrk was not 100%, but about 70 and 65% in 3- and 5-day females, respectively (Figure 2C). Our results suggest that pburs acts through Tcrk and possibly IIS/TOR signaling to influence reproduction (Figure 8E).

## Discussion

In this study, we explored the role of burs homodimer and its receptor Tcrk in regulating the reproductive physiology of *T. castaneum*. Because the expression profiles of *Vg* and *VgR* are often used as indicators to judge the reproductive function of female insects (Tufail and Takeda, 2008, 2009), we first established a correlation between the expression profiles of burs and pburs and those of *Vg1, Vg2*, and *VgR*, i.e., the expression peaks of *burs* and *pburs* ahead of the expression of *Vg1, Vg2*, and *VgR* (Figure 1), suggesting that burs and pburs may influence *Vg1, Vg2*, and *VgR* expression in *T. castaneum*. We then investigated the effect of burs, pburs, and Tcrk RNAi on the expression of *Vg1, Vg2*, and *VgR*. The knockdown of burs, pburs, or Tcrk significantly downregulated the expression levels of *Vg1, Vg2*, and *VgR* (Figure 2), suggesting that burs, pburs, and Tcrk are involved in the reproductive process. Consequently, burs or pburs RNAi led to the abnormal developmental pattern of oocytes (Figure 3) with limited Vg content (Supplementary Figure 3 and Figures 4E,F), decreased egg number, and reduced egg hatch rate (Figures 4A,B). Because burs silencing completely blocked egg hatch, we used pburs RNAi to investigate the phenotype of the offspring. Knocking down of pburs led to smaller larvae, prolonged developmental period, and reduced pupation rate. The knockdown of pburs also affected female pupal size, adult eclosion rate, and offspring female fecundity (Figure 5). Additionally, the expression levels of *pburs* in offspring larvae and females were also decreased compared to the control group (Supplementary Figure 4), indicating that the effect of pburs RNAi could be inherited in the next generation in *T. castaneum*. RNAi inheritance has been shown in *Caenorhabditis elegans*, and in some cases, can persist for more than five generations (Vastenhouw et al., 2006). The long persistence and parental transmission of RNAi have also been demonstrated in the triatomine bug, *Rhodnius prolixus* (Paim et al., 2013). Similarly, recent studies have shown that in the rice leafroller, *Cnaphalocrocis medinalis*, after the knockdown of *CmHK* in parental adults, egg production and hatchability of generation (G) 1 and G2 both were inhibited (Shakeel et al., 2020). These results proved that RNAi could cross insect generational boundaries. In addition, our results are consistent with these reports that the RNAi effect of pburs can be sustained in the next generation.

Vitellogenesis in *T. castaneum* is regulated by JH and nutrition-related IIS/TOR pathways (Parthasarathy et al., 2010b; Parthasarathy and Palli, 2011; Sheng et al., 2011). In this study, burs or pburs RNAi downregulated the *TOR* downstream target genes *SK61* or *SK62* and upregulated the expression of the TOR pathway inhibitor *4EBP* (Figures 6A–D). Simultaneously, burs and pburs could mediate the expression of the IIS pathway receptor *InR* and the main factor *Akt* (Figures 6E,F). All of these IIS/TOR pathway components have been shown to affect *Vg* gene expression and Vg protein biosynthesis in insects (Roy et al., 2018). Our results also showed that burs or pburs RNAi could downregulate the expression of *Tai* (Supplementary Figure 5C), indicating that JH and IIS/TOR signaling may be involved in burs and pburs-mediated reproductive physiology in *T. castaneum*.

We then expressed and purified r-pburs to investigate whether pburs was able to induce the expression of *Vg1, Vg2*, and *VgR* in previtellogenic females. Apparently, r-pburs significantly enhanced the expression of these three genes (Figures 7E–G), a result consistent with the report by Sathapondecha et al. (2015) that bursicon significantly increase *Vg* expression and ovarian development in the black tiger shrimp. In vertebrates, gonadotropins, such as follicle-stimulating (FSH), luteinizing hormone (LH), and chorionic gonadotropin, are also considered as a cystine knot protein consisting of alpha and beta subunits and have been shown to control gonad development (Burger et al., 2004). Moreover, FSH and LH can stimulate follicular development, steroid hormone production, and ovulation (Ulloa-Aguirre and Timossi, 1998; Weghofer et al., 2007). In addition, gonadotropins are proven to work through their specific receptor, leucine-rich G protein-coupled receptors (LGR) (Hearn and Gomme, 2000). Furthermore, gonadotropins-like hormones have been identified in crustaceans and indicated to be involved in gonad-stimulation development as the levels of FSH-like peptides increased in the vitellogenic stage of ovarian development (Ye et al., 2011). These suggest that burs, as a cystine knot peptide, may also function in reproduction of arthropods.

In addition, the r-pburs protein was able to significantly increase the expression of *S6K1, S6K2, InR*, and *Akt* (Figures 7I–L) and the results are consistent with the RNAi results, i.e., these genes were downregulated in pburs RNAi beetles (Figures 6B,C,E,F). Interestingly, the mRNA levels of JH-related genes, *JHAMT, Met*, and *Tai*, increased significantly as early as 6–12 h post-r-pburs injection (Supplementary Figures 5E–G). Previous research has shown that in *T. castaneum*, the injections of JH analog could not increase the *Vg2* mRNA level in InR or Akt knockdown insects (Sheng et al., 2011), but could indeed induce the expression of *Vg2* in the control group (Sheng et al., 2011), suggesting that JH might regulate vitellogenesis through the IIS/TOR pathway in *T. castaneum*. Our results showed that, in general, the expression of r-pburs-stimulated JH synthesis and signaling-related genes, such as *JHAMT, Tai*, and *Met*, are prior to or simultaneously with the expression of *S6K1, S6K2, InR*, and *Akt* in the IIS/TOR pathway. Therefore, we infer that bursicon homodimers may regulate the IIS/TOR pathway by influencing the JH pathway, consequently mediating reproduction in *T*. *castaneum*.

To confirm RNAi results, we performed rescue assays in pburs and Tcrk RNAi females using r-pburs. As expected, r-pburs was able to fully rescue the pburs RNAi effects by increasing the expression of *Vg1, Vg2*, and *VgR* (Figure 8A), as well as egg production (Figure 8C). However, r-pburs was not able to rescue the gene expression and egg production in Tcrk RNAi beetles (Figures 8B,D), suggesting that pburs act *via* Tcrk to regulate reproduction. Therefore, we infer that pburs regulate the expression of *Vg, VgR*, and JH and IIS/TOR pathway genes *via* Tcrk, thereby regulating reproduction in *T. castaneum*. Our hypothetical signaling pathway model of bursicon that regulates *Vg* expression in *T. castaneum* is shown in Figure 8E.

Our study showed that burs and pburs mediated the expression of *Vg* and *VgR* genes *via* Tcrk in *T. castaneum* possibly by regulating JH and IIS/TOR pathway-related genes, including *S6K, InR, Akt*, and *4EBP*, consequently affecting ovarian development, which ultimately influences female reproductive capacity. According to previous studies, burs homodimers work through DLGR2 to prevent midgut cell proliferation (Scopelliti et al., 2014) and regulate energy metabolism (Scopelliti et al., 2016, 2019). Moreover, impaired burs/DLGR2 signaling exacerbates glucose oxidation and depletes energy stores (Scopelliti et al., 2019), thus impacting the nutritional sources required for growth and reproduction, eventually leading to retarded larval development and reproduction in adults. Although r-pburs protein is able to rescue the pburs RNAi effect on the *Vg* gene expression and egg production and upregulate the expression of *Vg, VgR, S6K, InR, Akt, JHAMT, Tai*, and *Met*, we cannot exclude the possibility of the involvement of a heterodimer in regulating reproduction. In addition, how burs and pburs activate the JH signaling, and the IIS/TOR pathway remains to be explored at the protein and kinase level and demands further investigation. Nevertheless, our results reveal the key role of the neuropeptide bursicon homodimers in insect reproduction, thus providing insights for an understanding of the mechanisms of bursicon action in insects.

## Data Availability Statement

The datasets presented in this study can be found in online repositories. The names of the repository/repositories and accession number(s) can be found in the article/Supplementary Material.

## Author Contributions

JL and QS conceived the study. JL, ZZ, and JB conducted the experiments. JL drafted the preliminary manuscript. JL and ZZ interpreted the results. QS, BB, and QF refined and approved the final manuscript. All authors contributed to the article and approved the submitted version.

## Conflict of Interest

The authors declare that the research was conducted in the absence of any commercial or financial relationships that could be construed as a potential conflict of interest.

## Publisher's Note

All claims expressed in this article are solely those of the authors and do not necessarily represent those of their affiliated organizations, or those of the publisher, the editors and the reviewers. Any product that may be evaluated in this article, or claim that may be made by its manufacturer, is not guaranteed or endorsed by the publisher.
