# Supplementary material for: Neuropeptide Bursicon Influences Reproductive Physiology in Tribolium Castaneum
Source: Front Physiol. 2021 Oct 21;12:717437. doi: 10.3389/fphys.2021.717437 (PMC8567023; doi:10.3389/fphys.2021.717437)
Supplement: Supplementary file 1 [file Data_Sheet_1.pdf]

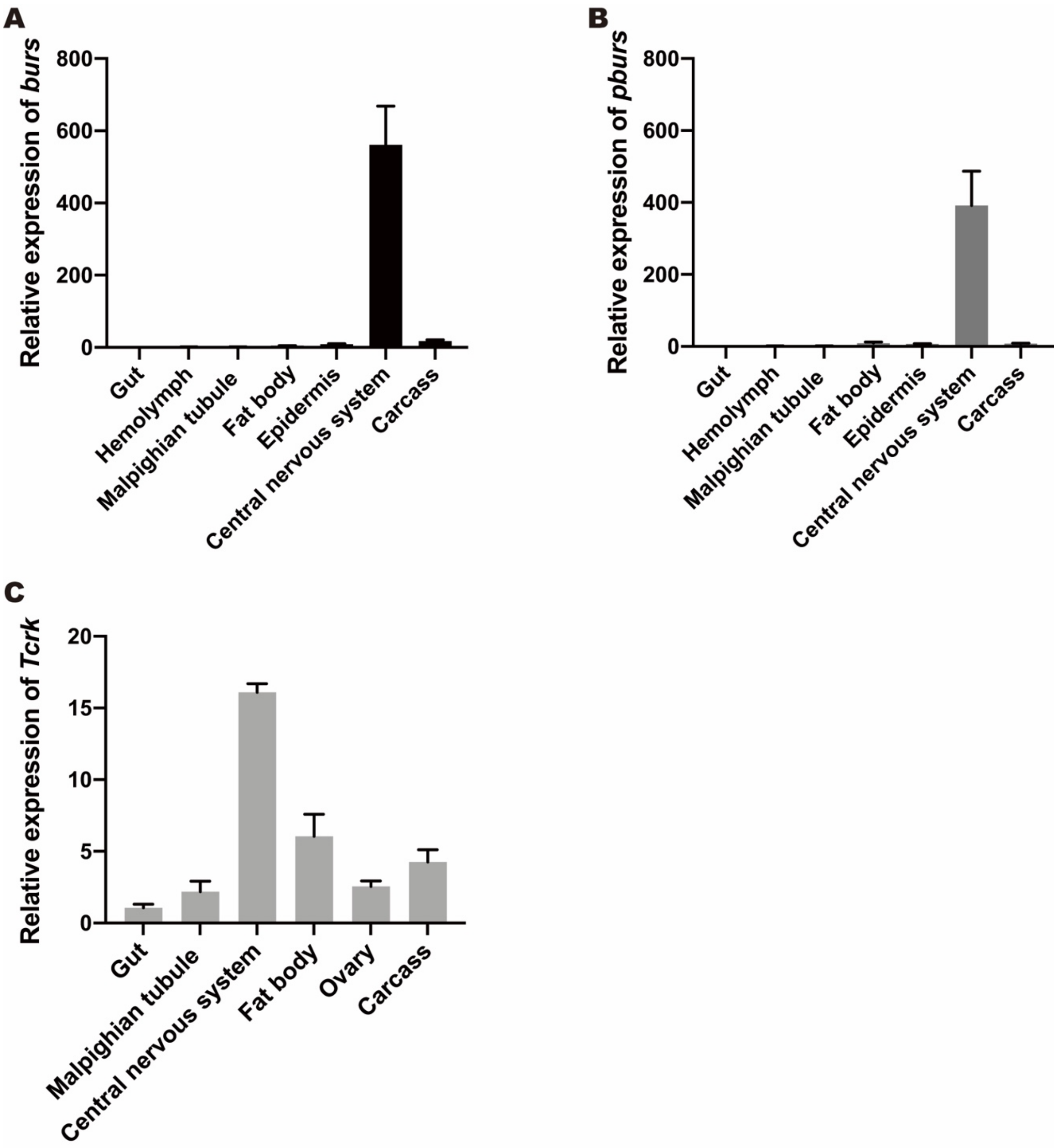

Supplementary Figure 1. Tissue distribution of **(A)** *burs* and **(B)** *pburs* in the 18-day *T. castaneum* larvae and **(C)** *Tcrk* in the 1-day *T. castaneum* female adults. The bars represent the mean ± SEM.

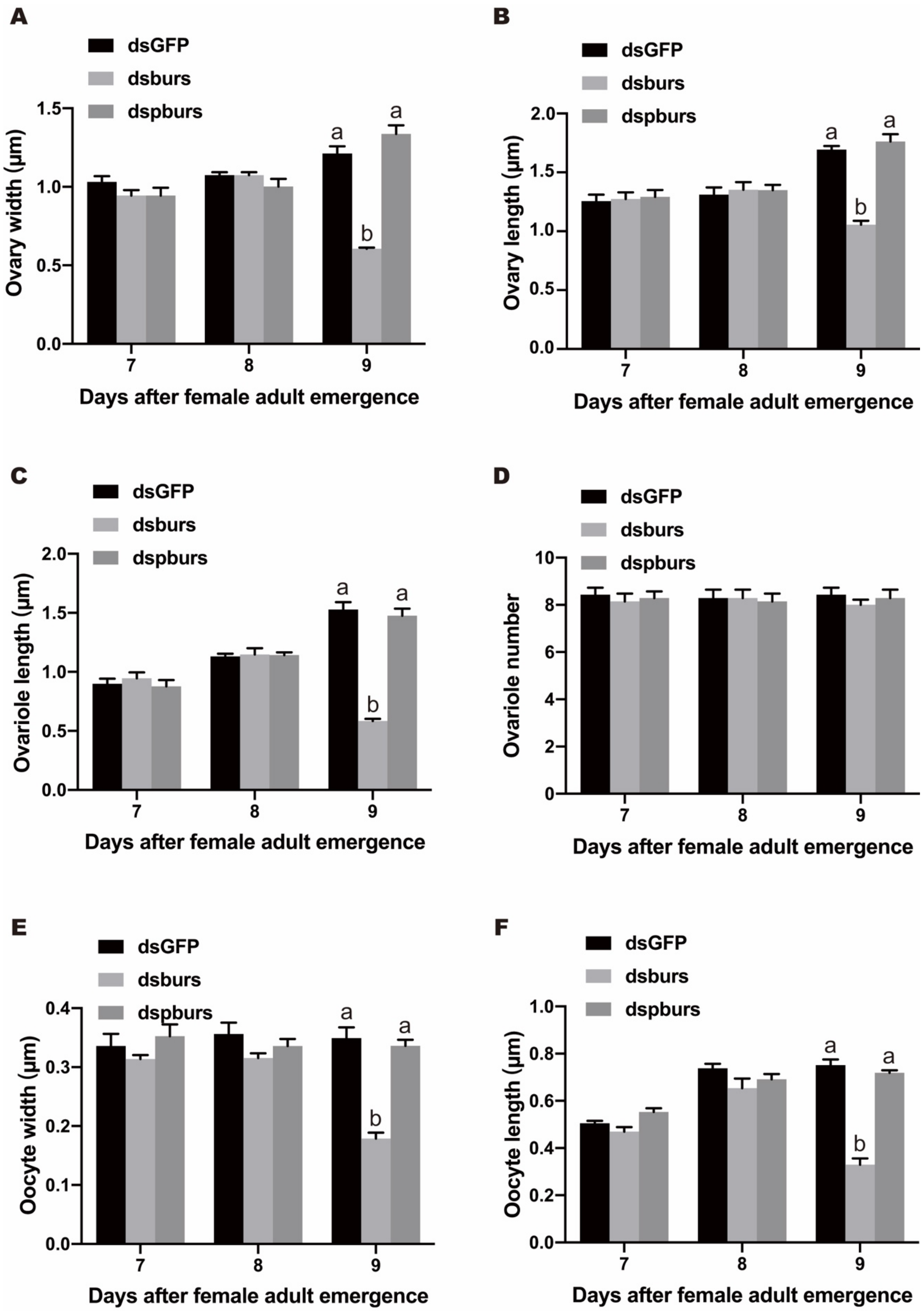

A
dsGFP
dsburs
dspburs
Ovary width (μm)
1.5
1.0
0.5
0.0
a
a
b
7
8
9
Days after female adult emergence
B
dsGFP
dsburs
dspburs
Ovary length (μm)
2.0
1.5
1.0
0.5
0.0
a
a
b
7
8
9
Days after female adult emergence
C
dsGFP
dsburs
dspburs
Ovariole length (μm)
2.0
1.5
1.0
0.5
0.0
a
a
b
7
8
9
Days after female adult emergence
D
dsGFP
dsburs
dspburs
Ovariole number
10
8
6
4
2
0
7
8
9
Days after female adult emergence
E
dsGFP
dsburs
dspburs
Oocyte width (μm)
0.4
0.3
0.2
0.1
0.0
a
a
b
7
8
9
Days after female adult emergence
F
dsGFP
dsburs
dspburs
Oocyte length (μm)
1.0
0.8
0.6
0.4
0.2
0.0
a
a
b
7
8
9
Days after female adult emergence

Supplementary Figure 2. Ovary, ovariole and oocyte size and ovariole number in dsGFP-, dsburs- or dspburs-treated female adults. **(A)** Ovary width, **(B)** ovary length, **(C)** ovariole length, **(D)** ovariole number, **(E)** oocyte width and **(F)** oocyte length of the 7-, 8- and 9-day females after dsGFP, dsburs and dspburs treatments. Different letters on the bars indicate the means ± SEM are significantly different ($p < 0.05$) among treatments by ANOVA.

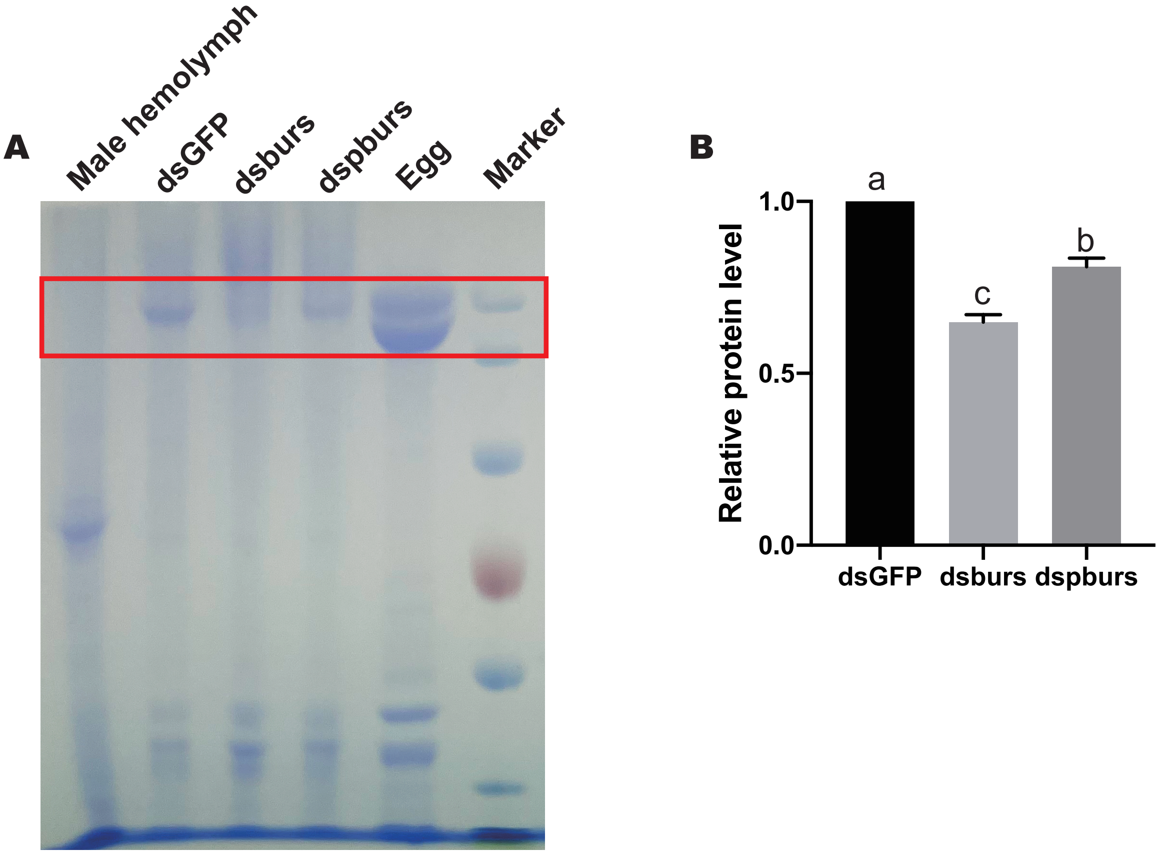

Supplementary Figure 3. (A) Coomassie Brilliant Blue-stained SDS-PAGE showing Vg protein in the 8-day female ovary after burs or pburs silencing. Male hemolymph: male hemolymph protein as negative control; dsGFP: protein from the 8-day ovary after dsGFP treatment; dsburs: protein from the 8-day ovary after dsburs treatment; dspburs: protein from the 8-day ovary after dspburs treatment; Egg: protein from the 1-day eggs as positive control; Marker: protein molecular weight marker. The red rectangular box indicates the location of Vg protein. **(B)** Quantification of Vg protein in **(A)**, different letters on the bars indicate the means ± SEM are significantly different ($p < 0.05$) among treatments by ANOVA.

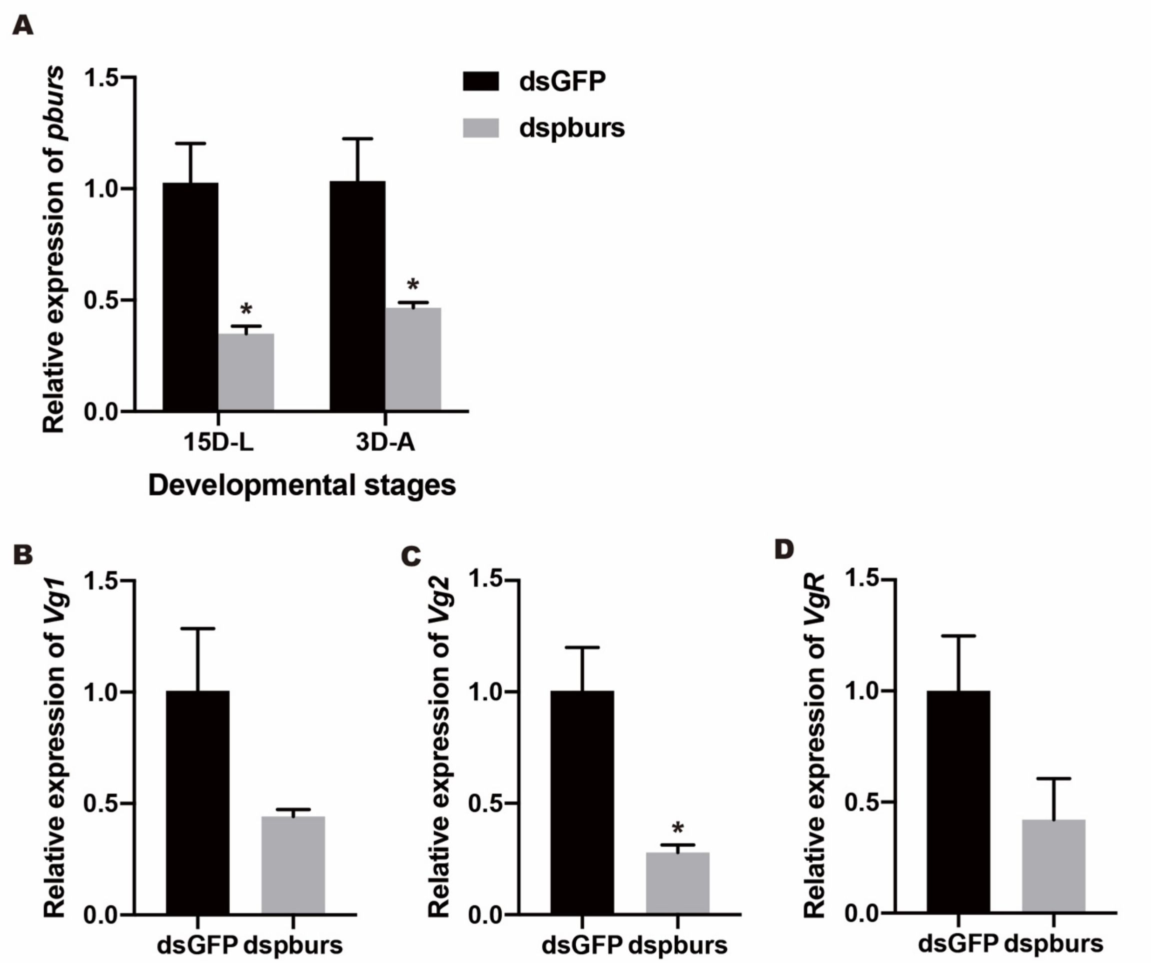

Supplementary Figure 4. Effect of dspburs treatment on the expression of *Vg*-related genes in offspring. **(A)** Relative expression of *pburs* in the 15-day offspring larvae and 3-day F1 female adults from the dsGFP- and dspburs-treated groups. **(B, C, D)** Relative expression of *Vg1*, *Vg2* and *VgR* in the 3-day F1 female adults from the dsGFP- and dspburs-treated groups. Asterisks above bars indicate significant differences in mean ± SEM between the treatment and corresponding control, $^{*}p < 0.05$ by *t* test.

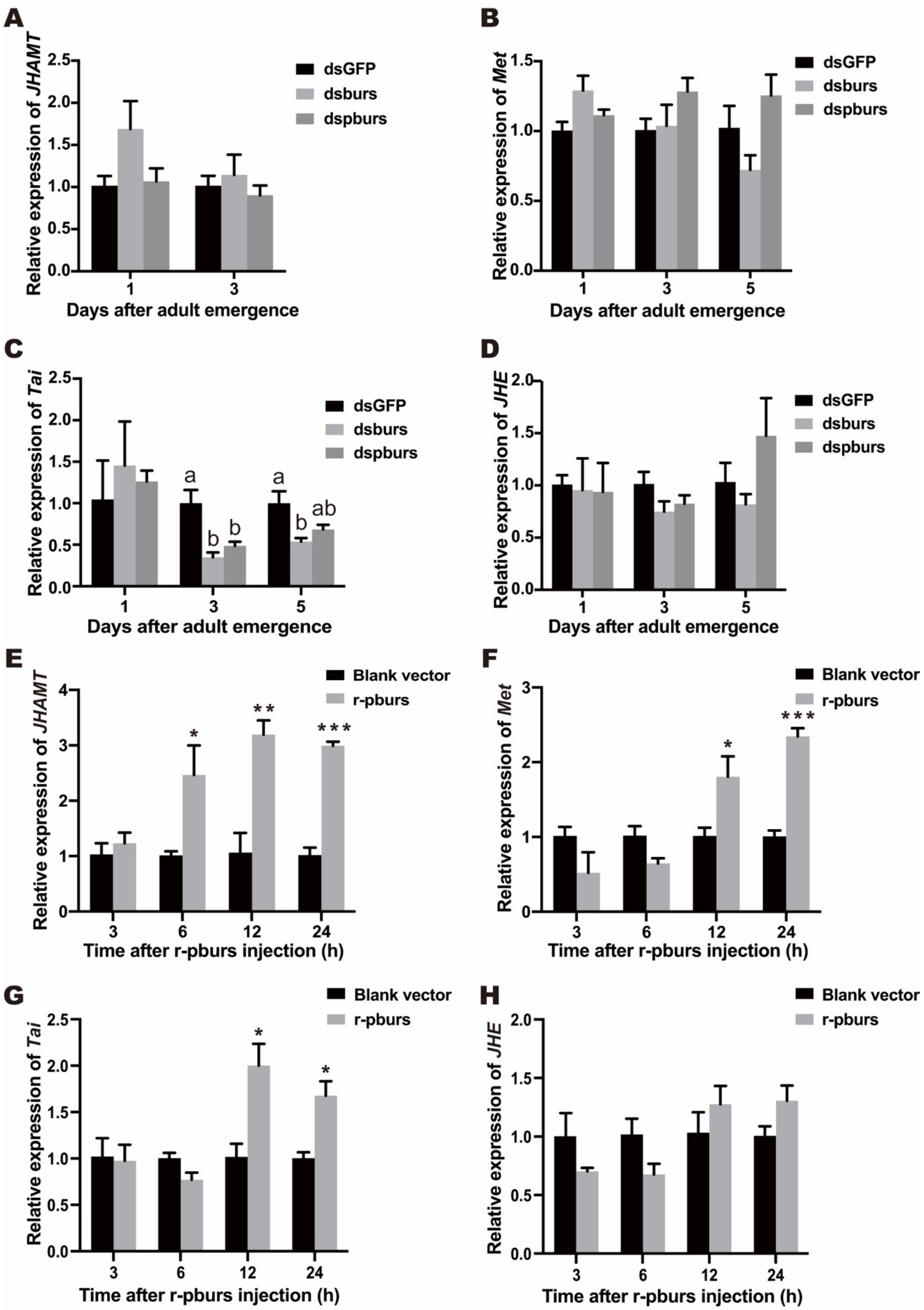
A
Relative expression of JHAMT
dsGFP
dsburs
dspburs
Days after adult emergence
B
Relative expression of Met
C
Relative expression of Tai
a
b
ab
D
Relative expression of JHE
E
Blank vector
r-pburs
Time after r-pburs injection (h)
F
G
H

Supplementary Figure 5. Effect of burs and pburs RNAi and r-pburs protein injection on JH-related genes. Relative expression of JH biosynthesis-related genes *JHAMT* **(A),** JH receptor genes *Met* **(B)** and *Tai* **(C),** and JH esterase gene *JHE* **(D)** in the 1-, 3- and 5-day female adults after burs or pburs RNAi. Relative expression of *JHAMT* **(E),** *Met* **(F),** *Tai* **(G)** and *JHE* **(H)** in females 3, 6, 12 and 24 h after r-pburs protein injection into the 1-day female adults. Different letters on the bars indicate the means ± SEM are significantly different ($p < 0.05$) among treatments by ANOVA. Asterisks above bars indicate significant differences between the treatment and corresponding control, $^{*}p < 0.05$, $^{**}p < 0.01$, $^{***}p < 0.001$ by *t* test.

Supplementary Table 1. Primers used for dsRNA synthesis, gene cloning and qRT-PCR analysis

Primer name	Sequence (5'-3')	Purpose
GFP-F	TAATACGACTCACTATAGGGTGGTCCCAATTCTCGTGGAAC	dsRNA synthesis
GFP-R	TAATACGACTCACTATAGGGCTTGAAGTTGACCTTGATGCC	
Burs-F	TAATACGACTCACTATAGGGGTGATCCACGTTTTACAATATC	
Burs-R	TAATACGACTCACTATAGGGTAGATAGAGGCCCTTCGTC	
Pburs-F	TAATACGACTCACTATAGGGACAGAGAATATGCAATGGGGAG	
Pburs-R	TAATACGACTCACTATAGGGGAGACGCACGCCGTCAGGG	
Tcrk-F	TAATACGACTCACTATAGGGCATGTTCATCAACGGAGTGG	
Tcrk-R	TAATACGACTCACTATAGGGTCTCTCGCCCAGGAGTTTTA	
Tcrp3-F	TCAAATTGATCGGAGGTTTG	qRT-PCR
Tcrp3-R	GTCCCACGGCAACATAATCT	
Burs-F	ACCGACGAGTGCCAGGTC	
Burs-R	ATTATTGAGAGTGAGACTTGAG	
Pburs-F	ATCAAAGAGGAATTCGACGAAT	
Pburs-R	TTATCGGCTGAAATCGCCAC	
Tcrk-F	ACCAGTATGCAGATGGAGGACGAA	
Tcrk-R	TTATCTGGAAGGCCCTGCTTCACT	
Vg1-F	TTGCAAATGCTGGGTGGTGAAGAC	
Vg1-R	AGCGTGTGCGTTGATAACTTGCTG	

Vg2-F	AACGCACACGATTTCGACCAAGTG	
Vg2-R	ACGGCAGCATTAACTTGGTTGCTC	
VgR-F	AGGTGACGTATTTGCTATCGCCCA	
VgR-R	AAGTGTCGCAATCCTCCTCTGGTT	
JHAMT-F	CATCTCGCCCTATCACCATTCG	
JHAMT-R	CCGCTGAAACCGATTTTGACAA	
Met-F	GGGAAAGCAAAGGATCATCA	
Met-R	AAGGCCTTCTTGCTCACTCA	
Tai-F	CAGAACGGAACAAAGACTCGG	
Tai-R	TGTTAATCTGCGCCTGAGGT	
Krh1-F	TGTGACGTTTGCTCGAAGAC	
Krh1-R	GCACGAGTAGGGCTTTTCAC	
JHE-F	CGAACCGCTGACTCCGTATTCA	
JHE-R	CTTCATCTTGGACACTACCCACCATC	
TOR-F	GAACGCACTGACACCAAACA	
TOR-R	CGGCAACTTTGGGATTCTAT	
S6K1-F	AGACGGGAAGCGATAAGGAAAGCA	
S6K1-R	TCAGCCTTAGTGTGTGCAGTGTCT	
S6K2-F	GCCTTTACGCCATGAAGGTGCT	
S6K2-R	CCACATCCACGAGTATGTTTCGCT	
4EBP-F	CCATCGCACTACTCCTCCAC	
4EBP-R	TGAAAACCGTCTGATGGGGG	
InR-F	CCTGGATTCGTTCAACAGGT	
InR-R	GATCGAGTTCACGAAGCACA	
Akt-F	CGACTTCACCAAGTGCAAAA	
Akt-R	GCCCCCTCATTGTAAACGTA	
Pburs-F	tagtccagtgtggtggaattcAAAATGTTCGACAAAATCATACTCTG	Gene cloning
Pburs-R	tcgaaccgcgggccctctagaTCGGCTGAAATCGCCACA	
